# Explanation

**Published:** 1855-03

**Authors:** 


### EDITORIAL DEPARTMENT.

---

*Explanation.*—In our account of the action of the Erie County Medical Society, we stated that a member had been previously expelled by simple resolution, for the practice of homœopathy, and that the courts sustained such action. The only importance attached to this statement is that it forms a precedent for the expulsion of J. D. Hill.

We gave what we supposed to be the facts in the case. On inquiry we find that the person alluded to, was declared *not to be a member*, by resolution. The resolution was based on some informality or illegality in his qualifications for membership, but the exciting cause of his expulsion was dishonorable conduct. He had previously been an active, and to all appearance, a legal member. Subsequent to his expulsion he became a quack.

This destroys our precedent, but a substitute is found in the expulsion by simple resolution, without explanation, of a Dr. Dean, for quackery, which occurred at about the same time. We have made this explanation because we would be guilty of no unfairness in our comments, but we do not recede from our expressed opinion that the action of the society at its recent meeting was legal, and demanded by good faith to its honorable members. H.

